# Comparing routine administrative data with registry data for assessing quality of hospital care in patients with myocardial infarction using deterministic record linkage

**DOI:** 10.1186/s12913-016-1840-5

**Published:** 2016-10-21

**Authors:** Birga Maier, Katrin Wagner, Steffen Behrens, Leonhard Bruch, Reinhard Busse, Dagmar Schmidt, Helmut Schühlen, Roland Thieme, Heinz Theres

**Affiliations:** 1 Berlin Myocardial Infarction Registry, Technische Universität, Berlin, Germany; 2 Department of Cardiology, Vivantes Humboldt Klinikum, Berlin, Germany; 3 Department of Cardiology, Unfallkrankenhaus Berlin, Berlin, Germany; 4 Department of Health Care Management, Technische Universitaet, Berlin, Germany; 5 Department Hospital Affairs, AOK Nordost, Berlin, Germany; 6 Department of Cardiology, Vivantes Auguste-Viktoria-Klinikum, Berlin, Germany; 7 Department of Cardiology, Jüdisches Krankenhaus, Berlin, Germany; 8 Department of Cardiology, Medical Park Humboldt Muehle, Berlin, Germany

**Keywords:** Health services research, Routine administrative data, Quality of care, Hospital performance, Myocardial infarction

## Abstract

**Background:**

Assessment of quality of care in patients with myocardial infarction (MI) should be based on data that effectively enable determination of quality. With the need to simplify measurement techniques, the question arises whether routine data can be used for this purpose. We therefore compared data from a German sickness fund (AOK) with data from the Berlin Myocardial Infarction Registry (BMIR).

**Methods:**

We included patients hospitalised for treatment of MI in Berlin from 2009-2011. We matched 2305 patients from AOK and BMIR by using deterministic record linkage with indirect identifiers. For matched patients we compared the frequency in documentation between AOK and BMIR for quality assurance variables and calculated the kappa coefficient (KC) as a measure of agreement.

**Results:**

There was almost perfect agreement in documentation between AOK and BMIR data for matched patients for: catheter laboratory (KC: 0.874), ST elevation MI (KC: 0.826), diabetes (KC: 0.818), percutaneous coronary intervention (KC: 0.860) and hospital mortality (KC: 0.952). The remaining variables compared showed moderate or less than moderate agreement (KC < 0.6), and were grouped in Category II with less frequent documentation in AOK for risk factors and aspects of patients’ history; in Category III with more frequent documentation in AOK for comorbidities; and in Category IV for medication at and after hospital discharge.

**Conclusions:**

Routine data are primarily collected and defined for reimbursement purposes. Quality assurance represents merely a secondary use. This explains why only a limited number of variables showed almost perfect agreement in documentation between AOK and BMIR. If routine data are to be used for quality assessment, they must be constantly monitored and further developed for this new application. Furthermore, routine data should be complemented with registry data by well-established methods of record linkage to realistically reflect the situation – also for those quality-associated variables not collected in routine data.

## Background

Collecting primary clinical data is still considered the gold standard for assessing health-care quality [1]. It is, however, an elaborate and expensive approach, especially if reliable and valid answers must be provided for various groups of patients with various diseases over relatively long periods of time. In recent discussions, it has consequently been argued that routine administrative data (e.g., claims data) may be used for quality assurance purposes if certain methodological standards are followed [2–4]. This could reduce costs and increase the reliability of data collected.

However, claims data show certain drawbacks implicit in the system if quality of care is to be assessed: i.e., they are primarily collected for reimbursement purposes and are only secondarily applied to assess quality of care [2, 5–7]. This situation gives rise to various consequences: e.g., administrative data lack essential quality assessment parameters [5]. They lack information essential for the patient's prognosis, i.e., whether a disease is present on admission (POA) or acquired in the hospital: indicators important in adjusting for differences between patients [8–10]. They also often show underestimation of comorbidities and risk factors unimportant for reimbursement [11–13].

In the German context there is, from a medical perspective, a lack of independent systematic analysis and controlled trials on the quality of coding in hospitals [5, 14]. It is argued that routine data should be used cautiously for quality assurance [15], and that misclassifications and non-standardized endpoints are common: a problem found not only in the German context [14–20]. Quentin et al. suggested for MI coding among 11 European countries [21] that reimbursement triggers ICD coding.

Despite these drawbacks, the use of administrative data for assessing quality of care in Germany is becoming more popular, quality of data is improving and various projects assessing quality of care on the basis of administrative data have evolved in the last years [22–24].

But the following questions remain: whether German claims data provide *valid* information on the quality of care provided, under which circumstances this is feasible and which data should be used [25]. These questions provided the basis of our study. As carried out previously in a French group that compared routine data on stroke with registry data [26], we used the data of a clinical quality assurance database (Berlin Myocardial Infarction Registry) to validate the administrative data of a large German sickness fund (AOK Nordost).

We chose acute myocardial infarction (MI) as disease entity, because MI is a substantial public health burden [27] with acknowledged guideline-based treatment strategies, extensive coverage by public reporting [2] and a uniform clinical data standard on acute coronary syndromes [28].

## Methods

### The Berlin Myocardial Infarction Registry

Our study was based on the long-term experience of the Berlin Myocardial Infarction Registry (BMIR), which works to improve the quality of hospital care for MI patients and has continuously collected data on the treatment of patients with MI since 1999 [29, 30].

All MI patients enter the registry who reach a hospital within 24 hours after symptom onset with Type I MI, according to the universal MI definition [31]. Specially trained physicians collect data by using a questionnaire that includes patient baseline characteristics, diagnostic and therapeutic measures taken during in-hospital course of events and discharge medication. After discharge, the questionnaires are forwarded to the BMIR Scientific Office at the Technische Universität Berlin, where entries are double-checked for errors and inconsistencies. To become a member of the BMIR the hospitals have to formally agree to include all MI patients Type I in the registry. Regular monitoring and peer review take place, and site audits are performed to ensure that patients are consecutively included in the registry. Patients are also selected randomly from every institution, with source-data verification for an average of 7.5 % of patients.

Patients’ data are collected pseudonymously. The registry was approved by the Berlin Board for Data Privacy Monitoring. The study protocol confirms to the ethical guidelines of the 1975 Declaration of Helsinki.

The BMIR is a self-organised institution run by cardiologists. Its structure promotes the internal validity of data collected, since those interested in participating in a *voluntary* registry do so out of self-interest and not out of fear of negative consequences [32] – and it provides clinicians a forum to communicate with each other regularly and to discuss problems jointly. This approach - as Bradley et al. were able to show - may reduce 30-day mortality rates in MI [33].

### AOK Nordost

In Germany, health insurance is mandatory for all citizens and permanent residents. It is provided mainly by competing, not-for-profit, nongovernmental health insurance funds (sickness funds) in the statutory health insurance scheme with a uniform benefit package – and by substitutive private health insurance for employees above an income threshold, civil servants and self-employed [34]. In 2011, 86 % of the population were covered by sickness funds and 13 % by private health insurance [35]. In 2011, 156 statutory sickness funds were registered in Germany. Since 1996, patients may choose which sickness fund they want to join. Large discrepancies exist among the sickness funds in regard to their structure and to the health care risks of their members. The AOK Nordost, one of the largest German sickness funds, insured 35 % of all MI patients treated in Berlin in 2009 – 2011 [27]. For historic reasons, patients insured with AOK are older, predominantly female, and are afflicted by more comorbidities than among the average German population.

German sickness funds collect data for reimbursement purposes through a system of diagnosis-related groups (DRG) [21]. Introduction of the DRG system in the German health care sector in 2004 – 2005 enabled for the first time the possibility of using routine data not only for reimbursement purposes, but also for assessing quality of care.

For claims purposes, physicians in German hospitals are legally obligated to code a principal diagnosis and an unlimited number of additional comorbidities or complications, in accordance with the German Modification of the International Classification of Diseases (10th revision). This enabled us to use ICD-10 I21 for defining patients with MI from the insurance data set, which reflects a diagnosis collected reliably in hospital settings as others were able to show [6, 36, 37]. We used the subcategories with a fourth character in I21.0 through I21.3. for defining patients with ST-segment elevation myocardial infarction (STEMI), and the remaining subcategories I21.4 and I21.9 for defining patients without persistent ST-segment elevation myocardial infarction (NSTEMI). This coding responsibility also enabled us to define patients’ risk factors and comorbidities using the following codes: hypertension I10, I11, I12, I13 or I15; smoking F17; hypercholesterolemia E78; diabetes E10, E11, E12, E13, or E14; renal failure N17, N18 or N19; congestive heart failure I50; atrial fibrillation I48.1; cardiogenic shock R57.0; stroke I60, I61, I62, I63, I64 or G45.

Physicians must likewise code operations and interventions according to the German Code for Operations and Procedures (OPS), adapted from the WHO International Classification of Procedures in Medicine. Percutaneous coronary intervention (PCI) as a procedure was operationalized with OPS 8-837 in our study.

No data on in-hospital medication are collected in the German system. Data on medication are routinely collected only in the outpatient setting. Pharmacies are reimbursed for the prescriptions handed out to patients. These data are coded according to the internationally accepted Anatomical Therapeutic Chemical Classification System (ATC). We used the following ATC codes for our analysis: beta-blockers as C07; ACE inhibitors and/or sartans as C09A, C09B, C09C, C09D or C09X; CSE inhibitors as C10AA, C10BA or C10BX; and antithrombotics as B01AC, B01AC06, B01AC04 or B01AC.

For our study we chose only those variables from the AOK data set that we assumed to be comparable to the variables collected in the BMIR, and that have been used by Freisinger et al. as well [38]. Other variables important for quality assessment in treatment of MI patients – i.e., time to intervention, guideline-recommended in-hospital medication, or smoking cessation counselling – were not available from the AOK data set.

For the purpose of our study, AOK patients’ data were processed pseudonymously. The Data Privacy Board of AOK Nordost as well as the Berlin Board for Data Privacy Monitoring agreed to the study and its design.

### Patients included

In our study we included all MI patients aged ≥ 20 years who were treated in a hospital in Berlin between 2009 and 2011. The *BMIR* included 9297 patients from 19 participating hospitals, of which 17 were equipped with a cath lab. From the AOK data, patients coded with the main diagnosis of ICD-10 code I21 were selected; they were 8909 patients from 37 hospitals, including 20 hospitals with a cath lab.

### Developing a data set for comparing documentation from AOK and BMIR

**Fig. 1:**
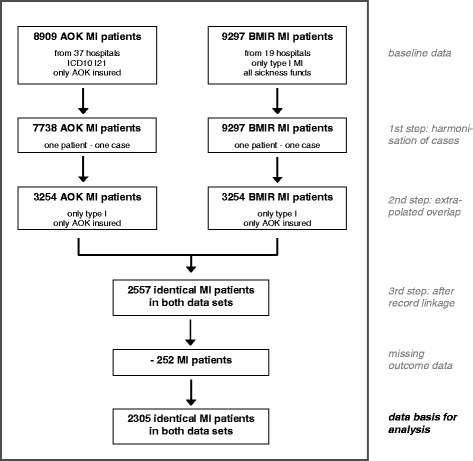
Flow chart: Developing a data set for comparing documentation between AOK and BMIR

To compare data from AOK and BMIR, we performed various methodological steps, which are summarized in a flow chart in Fig. 1.

#### 1st Step: Harmonisation of case definition

In comparison of data, case definition used in both data sets should be the same. In our first step, therefore, we harmonised case definitions from AOK and BMIR, with the BMIR definition as basis. The AOK system counts as one single case a transaction for which the hospital is remunerated, whereas the BMIR defines one patient according to a complete illness episode, including referral, transfer and readmission within 28 days [31]. AOK cases belonging to the same patient with the same illness episode were combined to represent one patient by joining them according to the point in time of treatment during the same illness episode. We applied this procedure to 13 % of AOK cases. We were left with 7738 AOK patients for further analysis, and with the problem of how to deal with differences in secondary coding among the various “single-case AOK patients”. We resolved this difficulty as follows: a “multiple-case AOK patient” was considered as suffering from hypertension, hypercholesterolemia, diabetes or stroke if any one of the single cases was coded with the respective disease; as suffering from ST-elevation MI or from a disease that could also be considered a complication of MI (atrial fibrillation, CHF, cardiogenic shock, or renal failure) if the chronologically initial case had the information for the respective disease coded; and as receiving PCI as first-line treatment if PCI treatment was coded in the chronologically initial case or in the direct-transfer case.

#### 2nd Step: Extrapolated overlap

Inclusion criteria differed for BMIR and AOK patients, which is a common phenomenon among different data sets, as also described by others [16, 17, 39]. To be able to compare both data sets, we had to define a possible overlap of patients from both data sets. Only patients with the classic spontaneous Type I MI based on atherosclerotic plaque rupture were entered in the BMIR. AOK included patients with all types of MI, since ICD-10 coding does not correspond to the typology proposed in the universal MI definition [31]. Type I MI, being the classic MI, is of primary interest with reference to consideration of treatment and outcome. Type II MI is considered a secondary MI with an imbalance between myocardial oxygen supply and/or demand, independent even of atherosclerosis. Treatment and prognosis of both types of MI differ, and recent publications show that patients with Type II MI comprise about one-fourth of MI patients and differ in clinical outcome [40, 41]. We therefore had to restrict our comparison to patients with Type I MI.

Since AOK included only patients insured by AOK (35 % of all MI patients), and since BMIR included all patients insured by any sickness fund or private health insurance plan, we had to restrict our analysis again to patients insured by AOK. Based on figures available for Berlin [27] and on the published distribution of MI types, we extrapolated a hypothetical overlap between the BMIR and AOK data to include 3254 patients (calculations not presented here).

#### 3rd Step: Record linkage

In a third step, we identified by record linkage those patients from the hypothetical overlap between the two data sets that were identical. Since data from both data sets were pseudonymized, we applied deterministic record linkage with 4 indirect identifiers for the linkage process [42, 43]: date (1) and time of admission in minutes (2), gender (3) and age (4) as key matching variables. Our aim in linkage was to provide a data set in which documentation for identical patients in both data sets could be compared, irrespective of the representativeness of the data and of the percentage of patients successfully linked. The record linkage process is described elsewhere [44]. We were able to successfully link 2557 patients, from which we had to subtract 252 patients. These were patients for whom the registry lacked outcome data because they were directly transferred to hospitals not participating in the Registry. We were left with 2305 matched patients as the basis for our comparison.

### Data analysis

We compared frequencies in documentation for linked patients from both data sets as percentages and calculated the kappa coefficient (KC) as a measure of agreement. A kappa coefficient < 0 was considered as no agreement, 0–0.20 as slight, 0.21 – 0.40 as fair, 0.41 – 0.60 as moderate, 0.61 – 0.80 as substantial and 0.81-0.99 as almost perfect agreement [36, 45].

## Results

After case harmonisation and record linkage, we arrived at a data set of 3205 linked patients for whom we compared documentation between the AOK and the BMIR data set.

The results showed that the *relative frequencies* of documentation between the two data sets varied. Documentation for some variables was comparable: e.g., 82 % treatment with PCI or 9.2 % in-hospital death. Certain risk factors – e.g., hypertension with 79.9 % in BMIR and 67.8 % in AOK, or smoking with 39.9 % in BMIR and 14.8 % in AOK and hypercholesterolemia with 49.5 % in BMIR and 41.8 % in AOK – were coded more often in the BMIR data. Other comorbidities – e.g., CHF with 39.3 % in AOK and 16.7 % in BMIR, renal failure with 23.8 % in AOK and 20.8 % in BMIR and atrial fibrillation with 15.5 % in AOK and 11.3 % in BMIR – were coded more often in AOK data.

The results also showed that the measure of agreement in documentation between AOK and BMIR ranged from almost perfect to hardly any agreement, depending on the variables compared: for example, 96 % of patients were treated in a hospital with a cath lab, according to both data sets, with a kappa coefficient of 0.902. On the other hand, the kappa coefficient was only 0.209 for documentation of CHF (for results see Tables 1, 2 and 3).

**Table 1:** Comparison of documentation of baseline characteristics of matched cases, with measurement of agreement

Comparison for matched patients	AOK (*n* = 2305)	BMIR (*n* = 2305)	*Kappa coefficient*	Classification according to categories
Age (mean)	69 yrs.		-	
Women	35 %		-	
Treatment in hospital with cath lab	96.5 %	96.6 %	*0.902*	I
STEMI	47.5 %	46.9 %	*0.835*	I
Diabetes	30.3 %	33.2 %	*0.819*	I
Hypertension	67.8 %	79.9 %	*0.417*	IIa
Smoking	14.8 %	39.9 %	*0.390*	IIa
Hypercholesterolemia	41.8 %	49.5 %	*0.341*	IIa
Previous stroke	0.9 %	9.7 %	*0.103*	IIb
Previous MI	3.2 %	24.8 %	*0.007*	IIb
Previous PCI	1.0 %	25.6 %	*0.010*	IIb
Atrial fibrillation	15.5 %	11.3 %	*0.582*	III
CHF	39.3 %	16.7 %	*0.209*	III
Renal failure	23.8 %	20.8 %	*0.618*	III
Cardiogenic shock	6.4 %	5.4 %	0.447	III

**Table 2:** Comparison of documentation of treatment and outcome of matched cases, with measurement of agreement

Comparison for matched patients	AOK (*n* = 2305)	BMIR (*n* = 2305)	*Kappa coefficient*	Classification according to categories
PCI (PCI for pts. coded as STEMI in AOK and BMIR) (PCI for pts. coded as NSTEMI in AOK and BMIR)	82.1 % 94.7 % 71.9 %	82.5 % 94.1 % 72.1 %	*0.903* *0.885* *0.925*	I
Hospital mortality	9.2 %	9.2 %	*0.979*	I
Length of stay in hospital (median IQR)	6 days (4/10)	6 days (4/9)	*0.868*	I

**Table 3:** Comparison of documentation for medication upon hospital discharge (BMIR) and medication over 2 quarters after discharge (AOK), with measurement of agreement

Comparison for matched patients	AOK (*n* = 2305)	BMIR (*n* = 2305)	*Kappa coefficient*	Classification according to categories
Statins	73.2 %	90.0 %	*0.281*	IV
ACE inhibitors and/or sartans	76.4 %	92.3 %	*0.192*	IV
Beta blockers	78.3 %	95.0 %	*0.166*	IV
Antithrombotics	81.0 %	98.5 %	*0.042*	IV

Since we observed such patterns of differences in reporting, we developed four categories to group the various variables studied (see also Tables 1, 2 and 3): one category for similar documentation in both data sets, a second category for less frequent documentation in AOK, a third category for more frequent documentation in AOK and a final category for different chronological documentation between the two data sets. Since mere frequencies are a poor measure of agreement, we extended our analysis to include the magnitude of the kappa coefficients in our categories. Using this approach, we arrived at the following categories:

**Category I** was defined as the category with data reported at the same frequency in both data sets and with almost *perfect agreement* between the two datasets (KC > 0.80). STEMI and diabetes as patient characteristics, treatment in a hospital with a catheter laboratory as a structural variable, treatment with PCI as a process variable, length of stay in the hospital and hospital mortality as outcome parameters were variables that fell into this category (Tables 1 and 2) and were considered valid and reliable data for assessing quality of care.

**Category II** was defined as the category with data reported *less frequently in the AOK* compared to the BMIR data set. This category was divided into two subgroups with subcategory IIa describing fair to moderate agreement between registry and sickness fund data (KC: 0.21 - 0.60). We found classic MI risk factors: e.g., hypertension, hypercholesterolemia and smoking in this category (Table 1). AOK data from this category were considered to be requiring caution in assessing quality of care. Subcategory IIb described hardly any agreement between BMIR and AOK data (KC: ≤ 0.20) and included previous diseases: e.g., previous stroke, previous MI and previous PCI. AOK data which fell into this subcategory were considered unusable for assessing quality of care.

**Category III** was defined as the category with data reported *more often in the AOK* compared with the BMIR data set. It was not necessary to form subgroups in this category, because all variables showed *fair to moderate* agreement between BMIR and AOK data (KC: 0.21 – 0.60). This category included classic MI comorbidities: e.g., renal failure, atrial fibrillation, congestive heart failure and cardiogenic shock. AOK data that fell into this subcategory were considered as requiring caution in assessing quality of care.

**Category IV** formed a special category on medication, because information on medication was collected at a different point in time in the two data sets. Category IV showed data reported *less frequently in the* AOK compared to the BMIR data set with in general documentation of a high number of patients receiving guideline-recommended medication in both data sets, but with a low kappa coefficient.

## Discussion

Assessing quality of health care should be based on the most accurate measures available, since consequences of incorrect measurements can be enormous [2]. If we consider, for example, pay-for-performance schemes that depend on quality of care delivered, or on hospital rankings with public reporting, accurate data and analysis are essential. Quality measures should also be accurate to increase trust in the data. It is physicians and other health personnel that must change their behaviour if quality of care is to be improved. If data on quality of care does not reflect the situation accurately and realistically, then health personnel will mistrust the data, which in turn will counteract any quality assessment initiatives [32]. When using routine data to analyse quality of care delivered, we therefore must be sure that these data provide a reliable, valid and trustworthy picture of the situation.

Using routine data for assessing quality of care is not new in the German context. What is new in our study, to our knowledge, is our approach: We have directly compared data from one of the largest German sickness funds with data from a quality registry by linking individual patients and comparing documentation in both data sets for exactly the same patient. This is a unique approach with individual record linkage, which enabled us to show that shortcomings assumed for routine data from various settings [5–21] were confirmed in a direct comparison for the German context.

### Validity of AOK data for quality assurance

Only a limited number of variables showed a high degree of agreement. If our results from Category I were more generalized, one could argue that structural factors (e.g., availability of a catheter laboratory), process indicators as indicated by procedures reimbursed by the sickness funds and coded according to the OPS classification (i.e. PCI), and hard outcome parameters (e.g., hospital mortality and length of stay in the hospital) are routine data comparable to data collected in the setting of a registry primary aimed to improve quality of care in the German setting.

Apart from these variables, two other variables also showed almost perfect agreement: STEMI (respective NSTEMI) and diabetes. For diabetes, others have also revealed that agreement between different data sources was high [11]. This has important implications for risk adjustment, since patients with STEMI or diabetes are acutely and more severely ill and have a higher in-hospital death rate than those without these diseases. The almost perfect agreement in STEMI documentation also showed that codes I21.0 through I21.3 may be used as an indirect measure to approximate the number of cases with STEMI in the absence of direct measure of STEMI in routine data. This aspect requires further study, since it may suggest a more homogenous subgroup of MI patients that could be more easily compared among different routine data sets [37].

Variables on *classic risk factors* disclosed underreporting and only a fair measure of agreement in the AOK data. Since risk factors per se do not influence reimbursement for acute hospital treatment of MI, the results of our analysis were as expected and showed unreliable coding in the sickness fund data. This is in line with the publication by Lujic et al. and makes routine data an unreliable tool for consideration of risk factors [12].

Variables on patients’ *history* with hardly any degree of agreement pose a special problem, possibly specific for the German context. AOK data were collected on a case basis. To enable analysis of a patient’s medical history, various cases from the past must be linked to constitute one patient. This is a difficult task, since initial DRG coding was introduced only in 2004/2005, and historical analysis cannot extend back beyond this date. Second, joining cases to constitute one patient over a long period of time involves a data privacy issue not welcomed by data privacy authorities in and outside the sickness funds. Third, sickness funds do not have an internal information system based on patients rather than cases, which also creates a technical problem for the sickness funds to unite various internal sickness fund data bases. Only limited data on patients’ history are therefore available from routine data – the farther back in time, the more difficult it becomes to obtain reliable information on patients’ medical history.

Variables on *classic MI comorbidities* that worsen patients’ prognosis and that – coded as a secondary diagnosis – increase the reimbursement received for the patient were more frequently reported in the AOK data set, which is in line with expected results. This is because, if a diagnosis is relevant to reimbursement, it is assumed that this increases the reliability of coding [46, 47]. At the same time, the measure of agreement on classic MI comorbidities between both data sets was only fair to moderate, which can be explained by the difference in data collection. AOK data show all secondary diagnoses regardless of whether they were present on admission or had developed as a complication during hospital stay. BMIR data reflect only comorbidities POA necessary for adjusting for differences in patients’ mix. Without being able to differentiate between POA, sickness fund data have only a limited ability to adjust for differences in patient mix [8–10] to assess of quality of care.

Since in-hospital medication is not documented in the sickness fund data, we had designed our study to compare discharge medication with medication in the outpatient setting two quarters after discharge – and we had expected results to be similar. But, as our results showed, there was a discrepancy between hospital discharge medication and medication after discharge, which may be attributed to the different points in time of data collection. This also may reflect an actual discrepancy between hospital discharge medication and medication after discharge – as others have also shown [48, 49]. It may also lie, however, in the fact that sickness funds have only those data on out-patient medication that are submitted for reimbursement by a pharmacist. Medications taken by the patients but not reimbursed do not appear in the sickness fund data set. The kappa coefficient may have been low (even though levels of agreement were high), because it depends on marginal frequencies [45, 50].

Since routine German data lack variables on quality of care other than those described, our results prompt us to argue that German routine data should either be broadened to include POA indicators, time indicators and drugs given in hospital – or should be regularly linked with registry data to reliably assess care of MI patients in the future.

### Limitations

The reliability of the AOK data depended on the export of data carried out within the sickness fund. Although we carried out plausibility checks on the data and compared them with other similar data, we cannot exclude the possibility that problems in data export could have arisen within the sickness fund. Our study design also did not allow verification of the ICD diagnosis of I21 by AOK data. As others have shown, however, the coding of I21 in the hospital setting is quite reliable [6, 36, 37].

Our study was based on registry data, which have their own drawbacks [51, 52]. Since the emphasis of our study lay on the analysis of data after linkage, possible drawbacks of BMIR data were of minor importance. The emphasis of our study was on comparison of documentation in AOK and BMIR data. With this approach, variables not available in routine data, but important for assessing quality of care, were not considered for our analysis (e.g., intervention time in STEMI patients).

Our study referred to the German context and was based on German legal aspects of diagnosis coding and procedures that may not be applicable to other national settings.

## Conclusions

Administrative data are collected for reimbursement purposes. If used for quality assessment at all, they are applied for this purpose only secondarily, which explains a number of their drawbacks. We have grouped the data in four categories to represent the various types of agreement or disagreement between two data sets. From these categories only those variables grouped in Category I had an almost perfect degree of agreement between both data sets. All other variables, especially those necessary for risk adjustment, showed only moderate to less than moderate agreement in documentation between the two data sets. They are therefore not considered as reliable tools for assessing quality of care. If routine data are to be used for quality assessment, they must be constantly monitored and further developed for this new application [20, 53]: e.g., by introduction of POA indicators, time indicators and drugs given in hospital. Furthermore, routine data should be complemented with registry data by well-established methods of record linkage to realistically reflect the situation – also for those quality-associated variables not collected in routine data.

## Abbreviations

AOK: Allgemeine Ortskrankenkasse (name of the sickness fund)
ATC: Anatomical Therapeutic Chemical (Classification System)
BMIR: Berlin Myocardial Infarction Registry
CHF: Congestive heart failure
DRG: Diagnosis-related Groups
KC: Kappa Coefficient
MI: Myocardial Infarction
NSTEMI: without persistent ST-segment elevation Myocardial Infarction
OPS: Operation- und Prozedurenschlüssel (Code for Operations and Procedures)
PCI: Percutaneous coronary intervention
POA: Present on Admission (indicators)
STEMI: ST-segment elevation Myocardial Infarction
